# Clinical Implications of a One-hand Versus Two-hand Technique in the Silfverskiöld Test for Gastrocnemius Equinus

**DOI:** 10.7759/cureus.6555

**Published:** 2020-01-03

**Authors:** David A Goss, Joseph Long, Adam Carr, Kyle Rockwell, Nicholas A Cheney, Timothy D Law

**Affiliations:** 1 Orthopedic Surgery, Associates In Orthopedics and Sports Medicine, Dalton, USA; 2 Medicine, Ohio State University, Columbus, USA; 3 Orthopedic Surgery, McLaren Greater Lansing, Lansing, USA; 4 Family Medicine, Western Reserve Hospital, Cuyahoga Falls, USA; 5 Foot and Ankle Surgery, OrthoNeuro, Columbus, USA; 6 Family Medicine, Ohio University Heritage College of Osteopathic Medicine, Athens, USA

**Keywords:** gastrocnemius equinus, silfverskiold

## Abstract

Introduction

Isolated gastrocnemius equinus contracture has been associated with several foot and ankle pathologies within the literature. The Silfverskiöld test is commonly used to identify isolated gastrocnemius contracture, however, the proper technique for performing the test has been scrutinized. The purpose of this study was to determine if there is a clinical significance in the ankle dorsiflexion that is obtained when the examination is performed incorrectly with a single hand versus the correct two-hand technique.

Methods

Thirty consecutive new patients with conditions associated with gastrocnemius equinus were included in the study. The Silfverskiöld test was performed with a two-hand technique and a single-hand technique. The amount of dorsiflexion obtained with the knee in full extension was measured and recorded using an extendable goniometer for each technique, with the arms aligned with the fifth metatarsal and fibular head.

Results

The average amount of dorsiflexion that was obtained with the two-hand technique with the knee in full extension was 76.3°±4.2°. When the one-hand technique was utilized the average amount of dorsiflexion obtained with the knee in full extension was 88.4°±4.2°. This was found to be statistically significant (p<0.01).

Conclusion

This study demonstrates that if the Silfverskiöld test is not performed correctly, the diagnosis of an isolated gastrocnemius contracture could be underappreciated. Accordingly, it may be important to perform the test with two hands in order to neutralize the hindfoot, midfoot, and forefoot, so that the dorsiflexion motion is through the tibiotalar joint alone.

## Introduction

Isolated gastrocnemius equinus contracture has been associated with several foot and ankle pathologies, including plantar fasciitis, Achilles tendonitis, hallux valgus, metatarsalgia, and adult-acquired flat foot deformity within the literature [1-5]. The diagnosis of an isolated gastrocnemius contracture is made by careful examination of the gastrocnemius and soleus muscle complex. Nils Silfverskiöld first described a clinical test to identify and isolate equinus contractures of the gastrocnemius [6]. The proper technique for performing the Silfverskiöld test is detailed in Dr. Sigvard Hansen Jr.’s book Functional Reconstruction of the Foot and Ankle [7]. They demonstrate and describe the test to be with the patient seated and the clinician utilizing two hands to neutralize the foot. One hand is used to stabilize the hindfoot by grasping the calcaneus and holding it in neutral. With the second hand, the midfoot is locked into an anatomic position with the navicular aligned over the talus and the forefoot manipulated into plantar flexion or pronation. This creates a neutral hindfoot, midfoot, and forefoot. Several other authors have highlighted the importance of stabilizing and neutralizing the subtalar and talonavicular joints in order to isolate tibiotalar motion to assess the gastrocnemius soleus complex [7-9]. Specifically, in a recent editorial, the proper technique for preforming the Silfverskiöld test was discussed, in which the clinical difference in ankle dorsiflexion that is obtained with and without the proper technique was brought into question [9]. As a result of that correspondence, we set out to identify if this variation in technique regarding the Silfverskiöld test is of clinical importance and found no current literature with data for this question. We hypothesize that there is a statistically significant difference in the amount of ankle dorsiflexion that is obtained when the examination is performed correctly with two hands and incorrectly with one hand.

## Materials and methods

Thirty consecutive patients with conditions associated with gastrocnemius equinus, such as plantar fasciitis, metatarsalgia, adult-acquired flatfoot, hallux valgus, Achilles tendonitis, and midfoot arthritis, were included in the study. Approval was obtained from the institutional review board. The data were prospectively collected and each patient was consented prior to inclusion in the study. 

**Figure 1:**
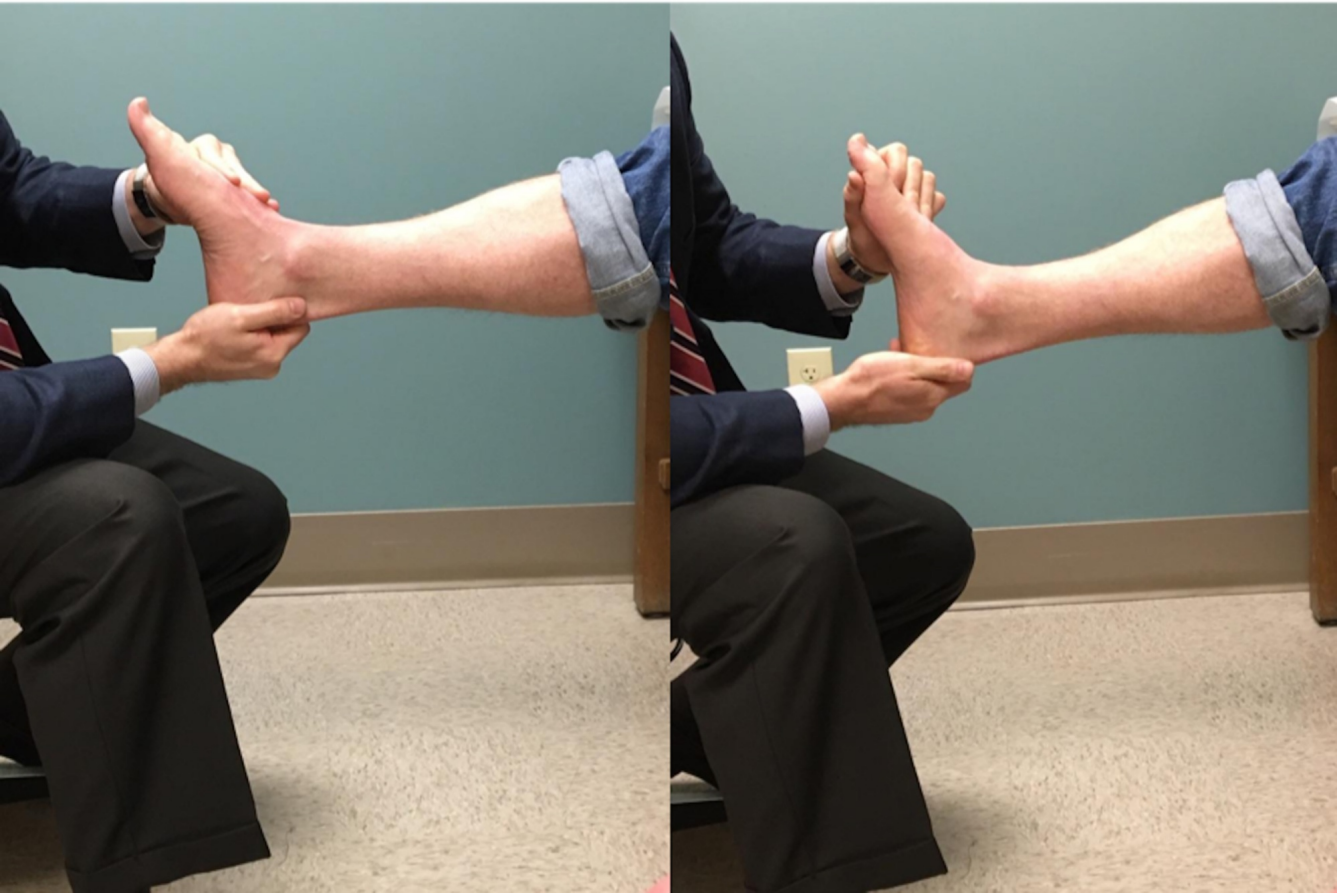
Correct demonstration of the Silfverskiold test The subtalar and talonavicular joints are locked in place in order to isolate motion through the ankle joint.

**Figure 2:**
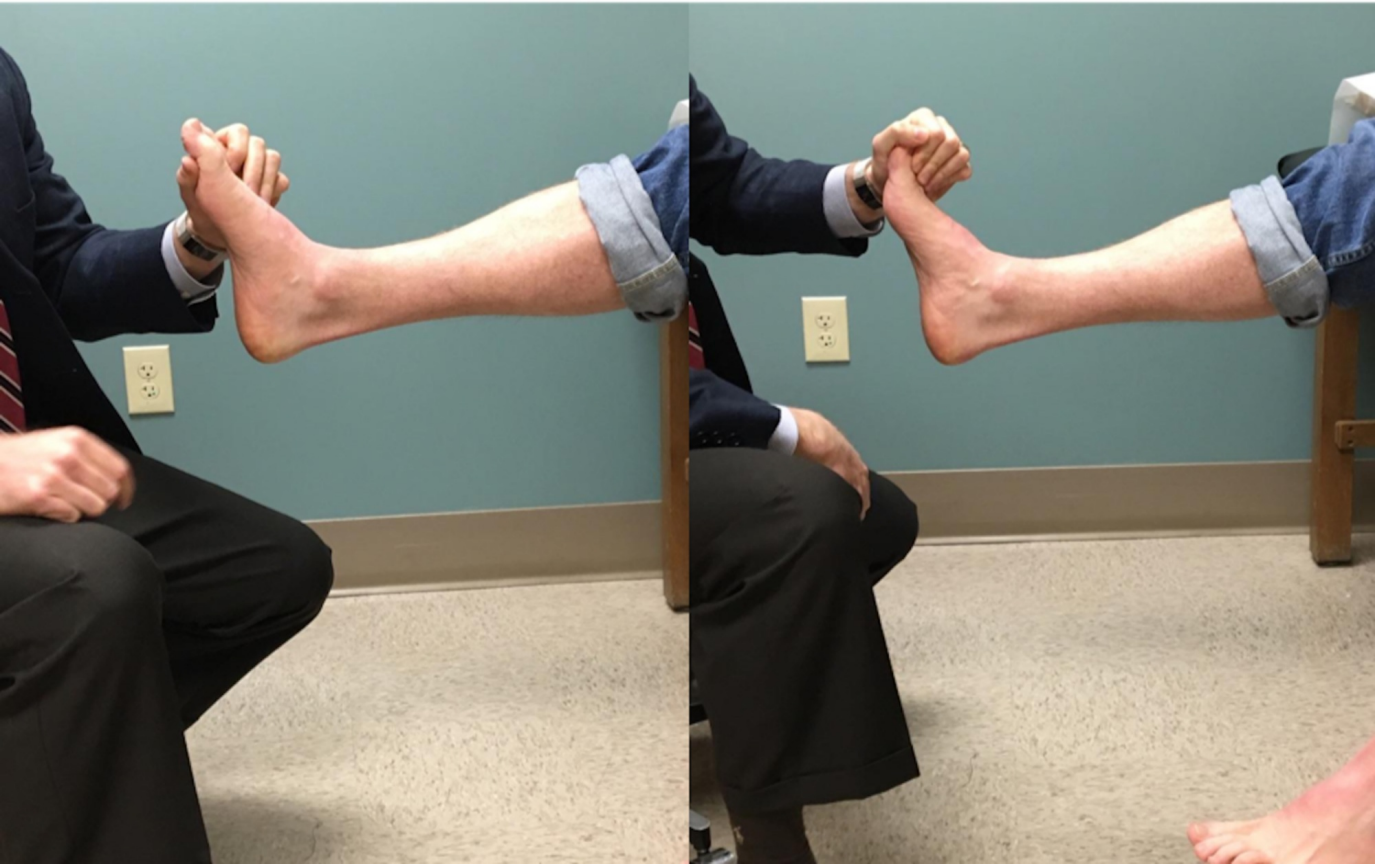
Incorrect demonstration of the Silfverskiold test Hind-foot and mid-foot joints are unlocked and free to travel through their respective ranges, causing an additive effect to the ankle joint.

The Silfverskiöld test was performed as part of the patient’s physical examination. The test was performed with the patient seated. Two hands were utilized to perform the technique, with one hand neutralizing and locking the subtalar (ST) joint and the other stabilizing the talonavicular (TN) joint and forefoot in order to isolate the ankle joint motion (Figure 1). We then performed the exam once again with the patient seated with one hand, without stabilizing the joints (Figure 2). A large extendable goniometer (Lafayette Instrument, Lafayette, IN) was used to measure the amount of dorsiflexion that was obtained with each test. The measurement was standardized by aligning the proximal arm of the goniometer with the long axis of the fibula, using the fibular head as a reference marker, and the distal arm with the long axis of the fifth metatarsal. The maximal amount of ankle dorsiflexion with the knee extended was measured and recorded. Zero degrees was defined as parallel to the long axis of the fibula with 90 degrees being perpendicular to the long axis of the fibula. The test was performed with the knee in full extension only. We set out to only evaluate the knee in extension in order to minimize the variables associated with taking measurements with the knee in flexion as well as to highlight the principles of the two versus one-hand technique. All measurements were performed once by the senior author and recorded.

## Results

**Figure 3:**
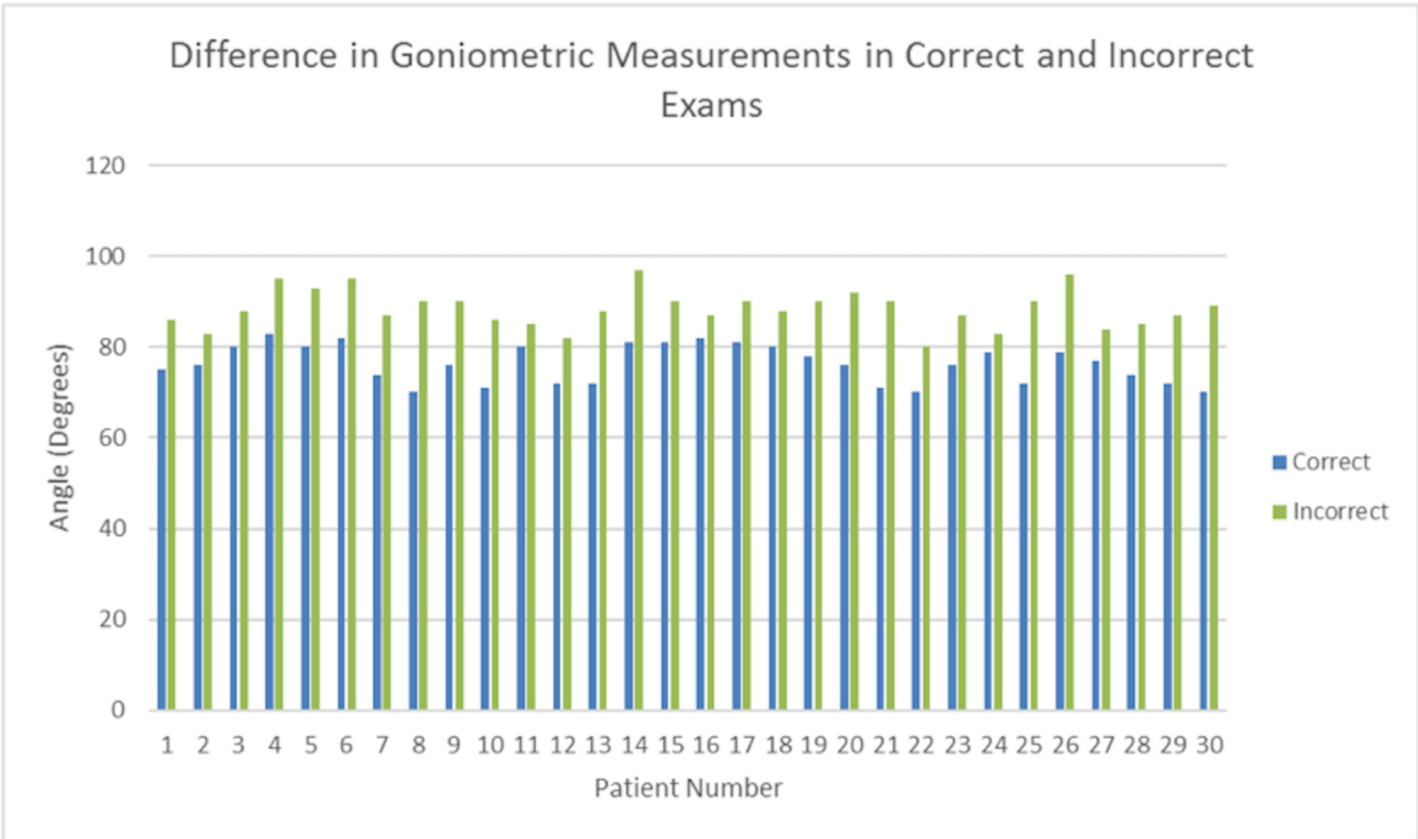
Bar graph comparing the correct and incorrect exams of each patient Each patient showed a greater degree dorsiflexion with the incorrect one-hand exam.

Thirteen patients were diagnosed primarily with plantar fasciitis, seven with transfer metatarsalgia, five with adult-acquired flat foot, three with hallux valgus, one with Achilles tendonitis and one with mid-foot arthritis. There were 25 females and five males, with an average age of 54.1 and 45.6 years of age, respectively. Eighteen extremities tested were left-sided and 12 were right. The average amount of dorsiflexion that was obtained with the two-hand technique and the knee extended was 76.3°±4.2°. However, when the one-hand technique was utilized the average amount of dorsiflexion obtained with the knee extended was 88.4°±4.2°. This was found to be statistically significant (p<0.01). Of note, all patients saw a decrease in the amount of ankle dorsiflexion when the test was done correctly, compared to when the test was done incorrectly (Figure 3).

## Discussion

We found that by utilizing the proper two-hand technique, there was a significant difference in the amount of ankle dorsiflexion obtained compared to a single-hand technique. When ankle motion was isolated by locking out the hindfoot, midfoot, and forefoot, there was 12.1° less ankle dorsiflexion. By improperly performing the Silfverskiöld test, it is conceivable that isolated gastrocnemius contractures are underappreciated or unrecognized. When a single-hand technique is used, dorsiflexion is gained through the ankle, hindfoot, midfoot, and forefoot, rather than isolating the tibiotalar joint.

There is some debate about how to accurately quantify an isolated gastrocnemius contracture and what exactly is a “positive” test. Some authors advocate that a positive test is when there is a 10° difference between the knee flexed and extended positions; however, some define it as an inability to dorsiflex past neutral [1,4,8]. While this debate is beyond the focus of this paper, it does need to be considered when analyzing our results. Regardless of how one defines a positive test, our study demonstrated that there is a significant difference in the amount of dorsiflexion obtained when the test was performed with a one-hand versus a two-hand technique, especially with 12.1° difference between the two techniques.

Several limitations existed with our study. Given that we utilized bony landmarks for our measurement points, it is possible that a morphological deformity to the fifth metatarsal or fibula could be a source of errant measurements. However, any patient with a history of previous foot or ankle fracture was excluded from the study. Another limitation of our study is that we are unable to draw any conclusions regarding what makes the Silfverskiöld test “positive” nor are we able to make conclusions about the clinical relevance of an isolated gastrocnemius contracture with this study.

## Conclusions

This study demonstrates that if the Silfverskiöld test is not performed correctly, the diagnosis of an isolated gastrocnemius contracture could be underappreciated. In these cases, patients may not be adequately diagnosed and their foot and ankle pathology may go untreated, leading to increased patient morbidity and lower patient satisfaction. Thus, our paper highlights the need to properly perform physical exam maneuvers in foot and ankle pathology, as the intricate biomechanics of the foot can lead to inaccurate findings.

## References

[1] Cortina RE, Morris BL, Vopat BG (2018). Gastrocnemius recession for metatarsalgia. Foot Ankle Clin.

[2] DiGiovanni CW, Kuo R, Tejwani N, Price R, Hansen S, Cziernecki J, Sangeorzan B (2002). Isolated gastrocnemius tightness. J Bone Joint Surg Am.

[3] Kiewiet NJ, Holthusen SM, Bohay DR, Anderson JG (2013). Gastrocnemius recession for chronic noninsertional Achilles tendinopathy. Foot Ankle Int.

[4] Maskill JD, Bohay DR, Anderson JG (2010). Gastrocnemius recession to treat isolated foot pain. Foot Ankle Int.

[5] Singh D (2013). Nils Silfverskiöld (1888-1957) and gastrocnemius contracture. Foot Ankle Surg.

[6] Nakale NT, Strydom A, Saragas NP, Ferrao PNF (2018). Association between plantar fasciitis and isolated gastrocnemius tightness. Foot Ankle Int.

[7] Hansen Hansen, Sigvard T (2000). Functional Reconstruction of the Foot and Ankle.

[8] Barske HL, DiGiovanni BF, Douglass M, Nawoczenski DA (2012). Current concepts review: isolated gastrocnemius contracture and gastrocnemius recession. Foot Ankle Int.

[9] Symeonidis P (2014). The Silfverskiöld test. Foot Ankle Int.

